# Editorial: Neurobiology, Clinical Course, and Therapeutic Approaches of Treatment-Resistant Schizophrenia: Toward an Integrated View

**DOI:** 10.3389/fpsyt.2019.00870

**Published:** 2019-11-21

**Authors:** Felice Iasevoli, Vincenzo De Luca, Frederick Charles Nucifora

**Affiliations:** ^1^Department of Neuroscience, University of Naples Federico II, Naples, Italy; ^2^Molecular Brain Science Department, Centre for Addiction and Mental Health, Toronto, ON, Canada; ^3^The Department of Psychiatry and Behavioral Sciences, School of Medicine, Johns Hopkins University, Baltimore, MD, United States

**Keywords:** psychosis, antipsychotic, clozapine, schizophrenia, treatment refractory

Treatment-resistant schizophrenia (TRS) is a disease entity whose tracts are yet to be fully deciphered. The characterization of effective therapeutic strategies for this severe condition represents one of the more relevant unmet need of contemporary psychiatry. Nonetheless, investigations on therapeutic strategies are strictly intermingled with the characterization of clinical determinants and diagnostic boundaries of the disease, and with the elucidation of its biological underpinnings. These elements cannot be separated from each other and their combined evaluation has been the objective of this Research Topic.

As an ideal introduction to the Topic, Leung et al. provide a thorough summary of the current knowledge on TRS. These Authors make an excellent overview of the current challenges with the definition and neurobiology of TRS, pointing out the heterogeneity of clinical course, the difficulty with an optimal characterization of predictors, and the lack of evidence based standard of care in TRS.

The idea that schizophrenia and TRS may be categorically distinct is tackled in the contribution by Kinon, as the Author critically discusses the issue of TRS heterogeneity. Recalling the classical definition of Bleuler for schizophrenia, Kinon proposes to refer to TRS as *The Group of Treatment Resistant Schizophrenias*, due to the patent heterogeneity in the trajectory of non-response to antipsychotics. This heterogeneity depends on multiple factors and mostly on inconsistency in defining TRS, preventing the possibility to understand whether TRS may be a distinct disease category or on a diagnostic continuum with schizophrenia. Parsing patient segments to achieve more homogenous ones sharing common pathophysiology may allow moving from more broadly to more targeted segments, paving the way to data or at least hypothesis-driven novel drug strategies for TRS.

In agreement with these reports, Iasevoli et al. has attempted to delineate the distinctive features and determinants of disease severity in TRS vs. non-TRS patients. We find that disease severity is higher in TRS patients and mostly associated with negative symptoms. In turn, negative symptoms mediate the effects of cognitive dysfunctions and are likely related to neurodevelopmental alterations in TRS patients. Despite this contribution appears to support the idea of a categorical distinction between TRS and non-TRS patients, it also enlightens one of the limitations of current operational criteria: the most relevant factor driving disease severity in TRS patients is the extent of negative symptoms, that are notoriously not targeted by current antipsychotics. A dog chasing its own tail.

These challenges and uncertainties strongly illustrate the urge to achieve pathophysiological models and neurobiological markers of TRS to develop targeted therapies. As reported in Leung et al. article, the traditional model of dopamine dysfunction for the pathogenesis of schizophrenia seems not to be applicable to explain TRS, and other neurochemical dysfunctions (e.g., cortical hyper-glutamatergy) may play a role in the disease.

In partial agreement with this consideration, the contribution of Amato et al. depicts an intriguing novel theoretical model to explain some forms of TRS. Based on previous experimental studies (1), Amato et al. suggest that response to antipsychotics may stem from an imbalance between D2 receptor blockade and dopamine transporter (DAT) blockade to achieve adequate extracellular dopamine levels to trigger presynaptic dopaminergic neuron autoinhibition. Presynaptic autoinhibition alleviates psychotic symptoms by reducing dopamine release and post-synaptic neuron activation. A failure of this mechanism, due to multiple factors (e.g., reduced DAT expression as a consequence of genetic factors, prior exposure to psychostimulants, or aging), may lead to treatment resistance.

Another remarkable contribution, by Mostaid et al., describes an overall upregulation of transcripts within the Neuregulin-ErbB signaling pathway among individuals with schizophrenia. Indeed, Authors investigated Neuregulin signaling pathway mRNA transcripts in whole blood of 71 TRS patients and 57 healthy controls and found upregulated levels in TRS patients for five transcripts, although only one surviving correction for multiple testing.

Still on neurobiological markers of TRS, the excellent review by MacKay et al. summarizes current findings on system and circuit-level brain dysconnectivity in treatment-resistant schizophrenia based on neuroimaging studies. As described in this report, a clear-cut separation at multiple levels of connectivity emerges between TRS and non-TRS patients, opening the way to circuit-based interventions.

The issue of therapeutic strategies has been addressed in multiple articles. An intriguing contribution is given by Miyaoka et al. These authors describe the case of a schizophrenia patient with predominant severe hallucinations and delusions non-responsive to antipsychotics, who showed a reduction of psychotic symptoms and improvement in social functioning after receiving bone marrow transplantation for acute myeloid leukemia. This case report has a place into the current debate on immune pathogenesis of schizophrenia (2).

Unfortunately, TRS patients are exposed to high doses of antipsychotics, causing severe undesirable effects. The contribution by Eriksson et al. deals with impaired bone mineral status, which was investigated in obese non-diabetic antipsychotic-treated patients, showing a reduction of bone mineral density in 23% of the subjects.

The search for strategies beyond mere pharmacological interventions is the object of the meta-analysis conducted by Polese et al. These authors focused on psycho-social interventions in TRS patients, either in augmentation or in substitution of antipsychotics. Psychological interventions showed a therapeutic effect in 40 of 42 selected studies. The most improvement was found in positive symptoms for cognitive behavioral therapy, as well as for other psychological interventions (albeit with different degrees). This contribution strongly encourages psychological interventions in TRS.

The contribution of Souto et al. illustrates the results of a randomized controlled trial for an online emotional training devoted to social cognition rehabilitation in schizophrenia patients. The authors found significant improvement in emotion recognition and multiple theory-of-mind tasks. Although to date impairment of social cognition has been only limitedly studied in TRS, it is presumable that social cognition-oriented interventions may soon become indicated in these patients.

However, literature on severe mental illness should face relevant methodological limitations, as illustrated in the contribution by Lally et al. The group found that psychotic participants in a large trial of psychosocial interventions to improve physical health in severe mental illness had a lower degree of overall illness severity and functional impairment than eligible non-participant psychotic individuals, therefore challenging representativeness of participants to the trial and concluding that more severe patients may tendentially be not predisposed to be enrolled. Although a generalization of these results to other kinds of trials (e.g., pharmacological or psychological) is beyond the authors' scope, more focused recruitment efforts should be considered when carrying out trials on severely ill patients. This recommendation should be specifically applied to TRS patients since they exhibit more severe psychopathology and more impaired social functioning even when compared to non-TRS patients (3).

## Author Contributions

The Editorial has been written by FI, VD, and FN.

## Conflict of Interest

The authors declare that the research was conducted in the absence of any commercial or financial relationships that could be construed as a potential conflict of interest.
